# German surgical residency training – quo vadis?

**DOI:** 10.1186/1754-9493-2-9

**Published:** 2008-04-25

**Authors:** Michael A Flierl

**Affiliations:** 1 Department of Pathology, The University of Michigan Medical School, 1301 Catherine Road, Ann Arbor, Michigan 48109, USA

## Patient Safety – A New Era in Surgery

Recently, patient safety has been featured in front-page headlines of major German newspapers and on news channels [1,2]. Triggered by the courageous "outing" of 17 well-established German physicians, disturbing rates of potentially preventable errors have been reported. Although the German "Aktionsbündnis Patientensicherheit" ("Action Alliance Patient Safety") was founded back in April 2005, it appears that the concept of patient safety has been "flying under the public radar" of recognition until present. Unfortunately, numerous prestigious German medical centers still consider adverse events, mistakes, and complications as a taboo, thereby preventing their own guild from learning from their mistakes and advancing patient care. In sharp contrast, the implemented standards for patient safety in the United States appear to be well ahead of the persistently anecdotal way of dealing with medical errors and complications in Germany [3,4]. Raising surgeon awareness for patient safety symbolizes medical innovation, and these advances start with medical education.

As a German medical graduate, I chose to pursue a postdoctoral research fellowship in the United States after graduation from Medical School. The rationale for this decision was to acquire a solid training in basic sciences before starting my clinical education as an Orthopaedic Surgeon. Currently, I have completed almost 3 years of basic research at a highly renowned academic institution in the United States, and I am thankful for the excellent experience in academic research and a successful initial publication track record [5-7]. Currently, I am at the crossroads of facing the major decision of whether to pursue my clinical training in Orthopaedic Surgery in Germany or in the United States.

## From Cutting-Edge Innovation to "Brain-drain"?

In recent years, the publicly feared "brain-drain" of experienced German physicians and young German medical graduates to more "attractive" countries, such as Switzerland, England, Australia, Canada or the United States has become an apparent reality [8]. In the German capital Berlin alone, 1,151 experienced physicians have recently left the country – a staggering 5% of all Berlin physicians [9]. How could the "German system" fall so low within such as short period of time? Only about 100 years ago, the field of surgery was dominated worldwide by innovative German surgeons, such as Theodor Billroth (1829–1894), Ernst Ferdinand Sauerbruch (1875–1951) or Gerhard Küntscher (1900–1972). In my opinion, Germany still produces outstanding surgeons and exceptional academic spirits. However, these potential pioneers are being largely oppressed and frustrated by a ramshackle medical system and a bloated, inefficient administrative apparatus. Thus, many innovative, free-spirited junior surgeons either fall prey to an apparently "deadbeat system", with little chance for advancement, or they choose to leave their country.

## The "German System" versus the "US System"

**Figure 1:**
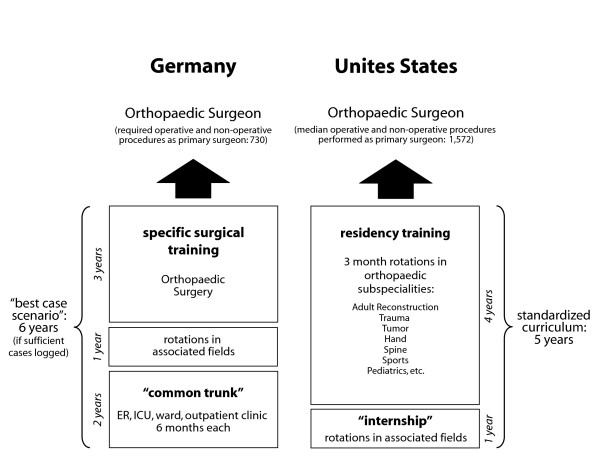
**Comparison of surgical residency training standards in Germany and the United States, as exemplified by the training of an Orthopaedic Surgeon.** Modified from [11, 12].

At this time, in the year 2008, there exist major differences between medical education and training in the United States and Germany. Examining these differences reveals the contrasts between a system that has evolved to nurture new ideas and talent and one that is moribund for lack of change. As outlined by Muensterer, the US-American concept has become one of the most innovative and highly dynamic medical training systems worldwide [10]. This superiority appears, at least in part, to originate from well conceived and standardized residency training programs. Due to a clearly defined duration of residency training, teachers are forced to convey their key messages to the residents and get the trainees ready for practice and board exams. This assures a high likelihood of graduation after a predefined number of years. In sharp contrast, the "German Medical Association" ("Deutsche Ärztekammer") solely defines a *minimal *duration of training; the key requirement is the recorded performance of a certain amount of operative and non-operative procedures, which usually takes significantly longer than the minimal duration of training (Figure 1). Under these premises, residents may have to attend their programs much longer than initially intended in order to become board eligible. While the average US resident performs 1,572 procedures during their five years of residency [11], German residents are eligible to be certified Orthopaedic Surgeons after performing just the minimal amount of 730 operative and non-operative procedures (Figure 1) [12]. Moreover, most German residents work under employment contracts which are limited to short periods of time, and thus often do not guarantee a complete residency training at a particular institution. Therefore, the "German system" lacks a solid foundation for effective training of clinical and surgical skills. Rather than reaching predefined goals during each postgraduate year and thereby increasing the individual responsibilities according to the corresponding level of training, these residents have to aim to "please" their superiors in order to obtain a contract extension. In contrast, US residents usually work under a guaranteed full duration of residency at a particular institution, which enables them to focus on their clinical training exclusively. Finally, German residents spend a significant part of their daily activities outside of clinical activities and out of a learning environment due to a massive burden of daily administrative and bureaucratic activities. In the US, most of these activities are performed by auxiliary services and physician assistants, leaving the physician with more time for essential duties, such as medical practice, teaching, learning, and, most importantly, patient care. Following each rotation, US residents evaluate their instructing attendings and vice versa, thus providing both sides with room for criticism and improvement. Evaluations are blinded and must be "360 degrees" with feedback from ancillary health care personnel, physicians and colleagues to insure as an objective sampling of critiques as possible. Each year, US residents have to undergo nationwide "mock" board exams which allow for evaluation of individuals and programs based on national averages thus providing a quality control for the didactic portion of resident training. American residency programs are scrutinized, updated and improved by the mechanism of standardized complete reviews every 3–5 years by a national, speciality-specific, Resident Review Committee (RRC). Not infrequently, programs with significant deficits are placed on probation. If improvements are not made within a set time frame, programs are closed. This is in sharp contrast to average German residency programs, which unfortunately still lack a standardized, periodical quality assessment of residency training.

"The good teacher's only concern is to teach his pupils how to succeed without him"

Andre Gide

(1869–1951, Literature Nobel Prize Winner 1947)

## Teaching – A Lost Art?

One of the key differences in the educational approach between US and German residency training is the recognition of the importance of teaching. In the US, second year residents will assist and teach interns, while being taught by senior and chief residents and attendings. In many US Medical Centres, surgical attendings mainly instruct and assist residents during cases. After all, surgery is a skilful craft, which can only be mastered by sufficient exposure, solid caseload and professional guidance by an experienced surgeon. Teaching in North America is considered an honour that is taken seriously. Through the evaluation process of faculty by residents, teaching physicians and their chairman receive direct feedback which is used to insure that educational goals are met. Those not performing appropriate supervision and teaching are often chastised by their departments and their status and salary may be adversely affected. Holding teaching physicians accountable for the performance of their residents develops a patient-centered culture as well as a positive incentive to educate effectively.

"Teaching is not a lost art, but the regard for it is a lost tradition"

Jacques Barzun

(*1907, French-American historian and educator at Columbia University)

Here is my current personal dilemma: the know-how in basic sciences which I have successfully acquired during three years of a postdoctoral research fellowship in the USA [5-7] provides me an excellent basis for a position as a resident in a renowned German academic medical center. However, under the circumstances outlined above, I fear that my clinical education and training of surgical skills will not be a departmental priority. As anecdotally illustrated by many other colleagues who are surgical residents at German University hospitals, it is more likely that my knowledge and experience in basic research would make me a candidate to represent the "motor" for the academic careers of more advanced residents and of selected attending surgeons in pursuit of their own habilitation thesis. For clarification, in Germany, the title of a "Privatdozent" ("Private Scholar") represents the prerequisite for being eligible for an academic promotion as a full professor and departmental chairman.

Based on my personal efforts to meet these expectations and hopes of more senior academic candidates, I may or may not receive a solid clinical training. The point for me and those in my stage of training is that once I am "accepted" into the German system, no matter how hard I am willing to work, I have no guarantee of becoming a trained and competent surgeon. Collectively, this brings me to what I believe to be the "key" problem: The true meaning of "University" – which represents a shortening of the Latin term "universitas magistrorum et scholarium" ("community of masters and scholars"), has been lost in German surgical residency training programs. No matter how well intentioned and idealistic my senior colleagues and mentors may be regarding surgical training, the existing system makes it nearly impossible for them to teach.

## Conclusion – "Should I Stay or Should I Go?"

I believe that US residency programs represent a highly structured, progressive and innovative educational system. The surgical caseload for US residents is more than doubled – within a shorter time-period of training – compared to the German numbers (Figure 1) and with a higher level of supervision by senior surgeons. Thus, by staying in the United States, chances are high that I will be a significantly better surgeon within a shorter time-frame, as opposed to returning back to my alma mater.

The new generation of surgical residents in the US is exposed to a "patient safety culture" from scratch, starting in medical school. The solid surgical proficiency after graduation further contributes to maintaining and enhancing patient safety goals. Germany is not only trailing the US in surgical residency education, but is also failing to shape new patient safety standards on a high, standardized level. As a German with a sense of pride regarding the tremendously innovative history of medical care in my home country, my great hope is that the ship will be righted so that we "expatriates" can learn, work and live at home. However, there is a long way to go and this "best case scenario" is by no means certain. The first steps of the long journey will begin with recognition and admission that we have a problem. The second steps will require examination of more effective and modernized training systems such as in the USA, in order to adopt best practices to German medical culture. If we, as German surgeons and surgical trainees, take these necessary actions, the final steps will be those of our best and brightest returning home to take care of our own.

## Competing interest statement

The author is a German medical graduate in search of a surgical residency training position.
